# High SLFN11 expression predicts better survival for patients with KRAS exon 2 wild type colorectal cancer after treated with adjuvant oxaliplatin-based treatment

**DOI:** 10.1186/s12885-015-1840-6

**Published:** 2015-11-02

**Authors:** Yanhong Deng, Yue Cai, Yan Huang, Zihuan Yang, Yang Bai, Yanlu Liu, Xiuping Deng, Jianping Wang

**Affiliations:** 1 Department of Medical Oncology, Gastrointestinal Hospital, Sun Yat-sen Universtiy, Guangzhou, 510655 China; 2 Department of Pathology, Gastrointestinal Hospital, Sun Yat-sen Universtiy, Guangzhou, 510655 China; 3 Department of Research Institute, Gastrointestinal Hospital, Sun Yat-sen Universtiy, Guangzhou, 510655 China; 4 Department of Colorectal Surgery, Gastrointestinal Hospital, Sun Yat-sen Universtiy, Guangzhou, 510655 China

## Abstract

**Background:**

SLFN11 was reported to be a predictive marker for DNA damage drugs. The study was to investigate whether SLFN11 expression is related to sensitivity to adjuvant oxaliplatin-based treatment in colorectal cancer.

**Methods:**

A tissue microarray, made with specimens from consecutive 261 patients who received oxaliplatin based adjuvant chemotherapy, was stained with anti-SLFN11 antibody. The staining was dichotomized as high or low expression. SLFN11 expression was correlated to clinicopathological factors, KRAS exon 2 mutation and survival.

**Results:**

SLFN11 high expression was found in 16.9 % of patients, and KRAS exon 2 mutation was detected in 32.2 % of patients. SLFN11 was expressed more common in well/moderate differentiation tumors(comparing to poor differentiation ones, 21 % v 4.9 %, *P* = 0.003) and stage II tumors(comparing to stage III tumors, 26.1 % v 11.4 %,*p* = 0.006). 23 out of 153 patients with *KRAS* exon 2 wild-type CRC had SLFN11 high expression, no death events was recorded in the 23 patients until last follow up. These patients had significantly better overall survival (OS) than those with SLFN11 low expression tumors (100 % vs 78.2 %, log rank *P* = 0.048). However, among patients with KRAS exon 2 mutant tumors, OS did not significantly differ between those with SLFN11 high and SLFN11 low tumors (Log rank *P* = 0.709).

**Conclusions:**

SLFN11 expression predicts good better survival in colorectal cancer patients with KRAS exon 2 wild type who have received oxaliplatin based adjuvant chemotherapy.

## Background

Genes of the Schlafen (SLFN) family are differentially regulated during thymocyte maturation. SLFN 1 in the T lineage profoundly alters cell growth and development. SLFNs have been linked to growth suppression, differentiation, and apoptosis [1]. SLFN11 belongs to group 3- SLFN family, and harbors a motif found in superfamily I DNA/RNA helicases [1]. SLFN11 expression was identified as the main correlate of sensitivity to irinotecan, a camptothecin analog that inhibits topoisomerase I (TOP1) [2]. SLFN11 expression is also very significantly correlated with response to Top2 inhibitors, alkylating agents, and DNA synthesis inhibitors, all of which are characterized as DNA-damaging agents (DDAs) [3]. DDAs are a mainstay of treatment for most human tumors. SLFN11 expression is causally associated with activity of DDAs in cancer cells [2, 3].

SLFN11 expression reportedly predicts independently overall survival (OS) in ovarian cancer patients treated with cisplatin-based regimens [3]. Oxaliplatin and cisplatin have a similar anti-tumor mechanism, which causes DNA damage. A range of SLFN11 expression in colorectal cancer (CRC) has been observed [3]. In this study, we investigated SLFN11 expression in stage II–III CRCs treated with oxaliplatin-based adjuvant chemotherapy (FOLFOX), and correlated it to clinicopathological characteristics and survival.

## Methods

### Patient cohort

Two hundred sixty one consecutive patients with high risk stage II or III colorectal adenocarcinoma treated between May 2007 and May 2012 were included in the study. All patients underwent quality assessed curative surgery and had received at least 3 month oxaliplatin based adjuvant chemotherapy in the Gastrointestinal Hospital of Sun Yatsen University. High risk factors for stage II disease included T4, vascular tumor invasion, poor differentiation, lymph node examination less than 12, and preoperative obstruction. This study was approved by the Medical Ethics Committee of Gastrointestinal Hospital of Sun Yatsen University. All patients gave informed consent for the use of tumor samples. Staging procedures, including colonoscopy, contrast enhanced CT scans of thorax, abdomen and pelvis and pelvic MRIs(rectal cancer), were performed in all cases to confirm locally advanced tumor stage and to exclude patients with evidence of distant metastatic disease at the initial diagnosis. Patients with family hereditary disease and multi-primary lesions were excluded.

### Treatment procedures

Pathological staging were determined according to American Joint Committee on Cancer (AJCC) guidelines. Adjuvant chemotherapy was started within 8 weeks after radical operation. Chemotherapy regimens contained mFOLFOX6(oxaliplatin 85 mg/m^2^ iv drip d1 + leucovorin 400 mg/m^2^ iv drip d1 + 5-Fluorouracil 400 mg/m^2^ iv d1 + 5-Fluorouracil 2400 mg/m^2^ CIV 46 h) or a few CAPOX(oxaliplatin 130 mg/m^2^ iv drip d1 + capecitabine 1000 mg/m^2^ bid d1-d14). Some rectal cancer patients received a total irradiation dose of 46 Gy in 23 fractions of 2 Gy with concomitant application of 5-fluorouracil (5-FU) prior to operation.

### Preparation of the tissue microarray

Paraffin blocks containing areas of invasive carcinoma were identified on corresponding H and E-stained sections. Areas of interest that represented the invasive front of the tumor, rich in non-necrotic tumoral glands, were identified and marked on the source block. The source block was then cored and a 1-mm core transferred to the recipient block using the Beecher Tissue Microarray (Beecher Instruments, Silver Spring, MD, USA). Two cores were arrayed per specimen.

### Immunohistochemistry staining and score

Tissue microarray slides were fixed in 10 % buffered formalin and embedded in paraffin. Sections were cut at 3 μm thickness, and mounted on coated glass slides. Slides were deparaffinized and heated. Sections were incubated with the primary anti-human SLFN11 antibody (1:50, Abcam). Negative controls underwent the same protocol with isoform antibody in a humidified chamber, and were refrigerated at 4 °C overnight. Incubated with polyperoxidase-anti-mouse/rabbit IgG (Zymed) for 15 min, 3,3′-diaminobenzidine was used as the chromogen. Immunostaining was evaluated by two independent pathologists blinded to the study. Agreement was achieved by discussion if there were discordance. Expression was analyzed by an individual labeling score considering percent of positive cells and staining intensity. Intensity was scored as 0, none; 1, weak; 2: moderate; 3, strong. The proportion of positive tumor cells was assigned to 0 (<25 % positive), 1 (25–50 % positive), 2 (50–75 % positive), 3 (75–100 % positive). The final score was calculated by intensity plus proportion (0–6).

## DNA preparation and KRAS mutational analysis

Formalin-fixed, paraffin-embedded tumor tissues were collected from Pathological tissue bank of Gastrointestinal Hospital of Sun Yat-sen University. All the tissue samples were confirmed independently by two gastrointestinal pathologists. Genomic DNA was extracted with the DNA FFPE tissue extracted Kit (BIOMIGA), according to the manufacturer’s recommendations.

The primers for the amplification and Sanger dideoxy chain termination sequencing of KRAS gene were forward: 5′- GTCCTG CACCAGTAATATGC-3′ and reverse: 5′-ATGTTCTAATATAGTCACATTTTC-3′ for codon 12 and 13. PCR was performed using 100 ng genomic DNA as template. Each mixture contained 10 pmol of each primer. The reactions were performed in a total volume of 31.45 μl. The amplification reaction were as follows: an initial denaturing cycle of 95 °C for 5 min; 45 cycles of 94 °C for 25 s, 58 °C for 25 s, 72 °C for 25 s; and a final extension cycle at 72 °C for 10 min. The PCR products were then purified and subjected to direct sequencing using the automatic sequencer (ABI-3730 Genetic Analysis, Applied Biosystems).

### Clinical evaluation and follow-up

All patients were reevaluated at 3-month intervals for 2 years and every 6 months thereafter. Evaluations consisted of pertinent medical history, physical examination, blood cell counts, and blood chemistry including carcinoembryonic antigen (CEA) levels at every follow-up visit. Colonoscopies (in patients treated with anterior resections) were performed at 6-month intervals in the first 2 years, and annually thereafter. Follow-up chest, abdomen and pelvis CT studies were scheduled every 6 months for the first 3 years, and annually thereafter. Histological confirmation of local recurrence and distant relapse (defined as tumor manifestation outside the pelvis) was encouraged. Alternate acceptable criteria included sequential enlargement of a mass in radiologic studies with simultaneous increase of serum CEA levels. Overall survival was defined as the time from surgery to detection of death. Median follow-up time was 49 months (range: 19–78 months). Follow up rate for this cohort was 95.6 %, 9 patients was lost during surveillance.

### Statistical analysis

Statistical analysis used SPSS 17.0. Frequency tables were analyzed using the *χ*^2^ test, with the likelihood ratio (LR) or Fisher’s exact test for categorical variables. Correlation of SLFN11 with clinical outcome variables was computed by Spearman rank correlation. ROC curve was used to set the cut-off point. Time to event data were visualized using Kaplan–Meier analysis with log rank test. Both univariate and multivariate survival analyses were carried out using Cox proportional hazard regression models. We estimated hazard ratios (HRs) with the fitted model coefficients and computed 95 % confidence intervals (CIs) and p values with Wald tests. P ≤ 0.05 was considered significant for all tests.

## Results

### SLFN11 expression status in CRC patients

**Fig. 1:**
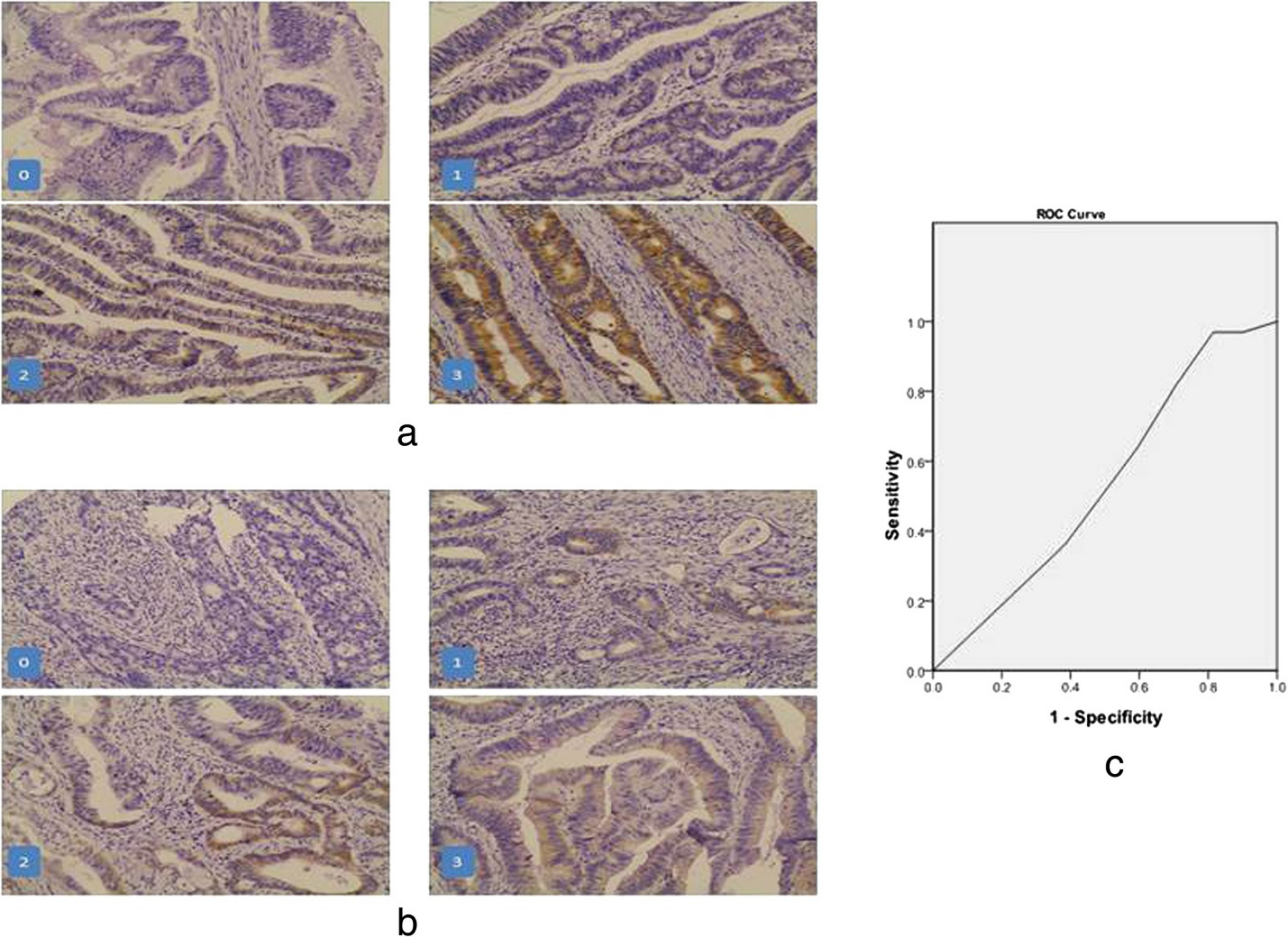
The typical staining of intensity: 0, none; 1, weak; 2: moderate; 3, strong (**a**). The typical staining of proportion: 0 (< 25 % positive), 1 (25–50 % positive), 2 (50–75 % positive), 3 (75–100 % positive) (**b**). ROC curve for SLFN11 expression score, the AUC = 0.526 (**c**)

The intensity and proportion of SLFN11 staining was shown in Fig. 1a and b respectively. SLFN11 expression was successfully obtained in 90.8 % (237/261) of CRC specimens tested. 237 patients were included in analysis for the following results. In a ROC curve for OS prognostic sensitivity and specificity of SLFN11 showed, the area under the curve was 0.526 (Fig. 1c) and the optimal cut-off was 4.5. We chose the cut-off point to dichotomize SLFN11 levels into high (>4.5) (SLFN11^high^) and low (<4.5) (SLFN11^low^). By this standard, 16.9 % (40/236) of patients tested had SLFN11^high^ tumors.

### Association of SLFN 11 expression with clinicopathological features

Table 1 shows patients’ clinicopathological characteristics. We then correlated SLFN11 expression with clinicopathological features of CRC, including primary tumor location, tumor differentiation, preoperative Cancinoembryonic Antigen(CEA) level, TNM staging, *KRAS* exon 2 status, age and sex. SLFN11 expression was expressed more in well/moderate differentiated tumors(compared to poor differentiated tumors, 21.0 % vs 4.9 %, *P* = 0.003) and stage II tumors(compared to stage III tumors, 26.1 % vs 11.4 %, *P* = 0.005).

**Table 1:** Patient clinicopathological characteristics and SLFN11 expression

		SLFN11Low	SLFN11High	P
Age (y)		55.72 ± 12.48	57.68 ± 9.98	0.351
Sex	Male	112	21	
	Female	85	19	0.727
Site	Left	43	4	
	Right	154	36	0.126
Grade	Well/Moderate	139	37	
	Poor/Undifferentiated	58	3	0.003
TNM stage	II	65	23	
	III	132	17	0.006
KRAS^a^	Wild type	136	23	
	Mutation	54	12	0.545
CEA(ng/ml)^a^		8.6 ± 17.57	10.34 ± 13.63	0.601

^a^ KRAS data was available in 225 patients, CEA data was available in 200 patients

### SLFN11 and survival

**Fig. 2:**
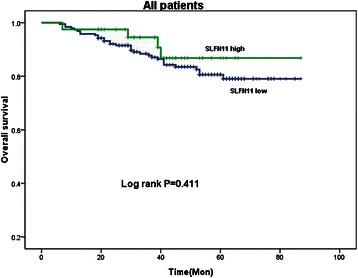
Log rank survival analysis showed no difference between patients with high and low SLFN11 expressing tumors in the cohort as a whole (Log rank *P* = 0.411)

The 3y-DFS and 5y-OS for the cohort was 78.9 % and 79.4 % respectively. Stage III patients have worse prognosis than stage II patients in terms of 3y-DFS(68.5 % v 88.2 %, HR 2.750, 95 % CI 1.424–5.311, *P* = 0.003) and 5y-OS(73.4 % v 89.5 %, HR 2.501, 95 % CI 1.199–5.217, *P* = 0.015), which was concordant with published data. Patients with SLFN11 high expression tumors tend to have better overall survival than those with SLFN11 low expression tumors. (86.8 % vs 80.6 %, HR 0.649, 95 % CI 0.229–1.837, Log rank *P* = 0.411, Fig. 2).

### SLFN11, KRAS and survival

**Fig. 3:**
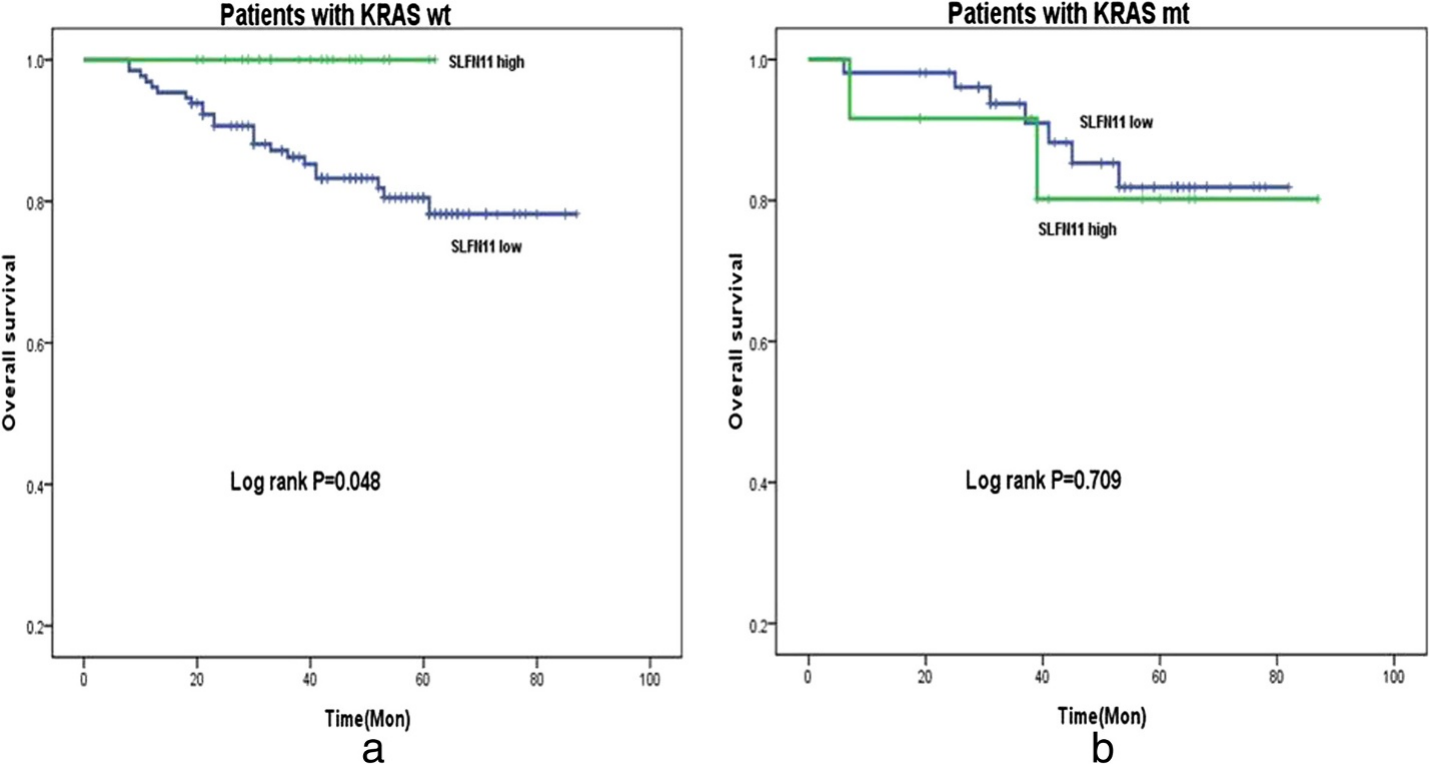
Among patients with *KRAS* wild-type tumors, those with high SLFN11 expression had significantly longer survival than patients with low SLFN11-expressing ones (Log rank *P* = 0.048) (**a**). However, among patients with mutated *KRAS* tumors, those with high SLFN11 expression had almost the same survival as patients with low SLFN11-expressing ones(Log rank *P* = 0.709) (**b**)

**Fig. 4:**
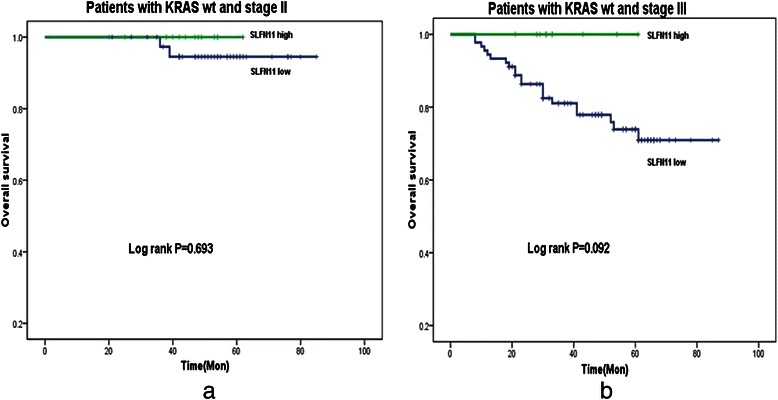
SLFN11 high expression patients had a trend of better survival than SLFN11 low expression patients both in stage II(**a**) and stage III(**b**) patients

Among 237 patients, 216 had available *KRAS* and survival data. The DFS and OS according to KRAS exon 2 status or SLFN11 expression status was shown in Table 2. In the cohort, 63 out of 216 had *KRAS* exon 2 mutated (*KRAS-mt*) CRC. OS for patients with *KRAS* exon 2 wild type (*KRAS-wt*) and *KRAS-mt* tumors was almost identical (80.6 % vs 82.8 %, Log Rank *P* = 0.823). If OS for the *KRAS-wt* group were analyzed separately from the *KRAS-mt* group in Kaplan–Meier analysis, overall survival curves of patients with SLFN11^high^ tumors and those with SLFN11^low^ tumors separated significantly (100 % vs 78.2 %, *P* = 0.048, Fig. 3a); the SLFN11^high^ group had longer survival. In contrast, in the *KRAS-mt* group, patients with SLFN11^high^ or SLFN11^low^ tumors did not significantly differ in OS (81.9 % vs 80.2 %, *P* = 0.709, Fig. 3b). Among 23 KRAS*-wt* and SLFN11 high expression patients, Only 2 recurrences were detected and no death was recorded. As SLFN11^high^ expression was more common in stage II. We did additional subgroup analysis according to the stage, in patients KRAS*-wt* tumors and found patients with SLFN11^high^ tumors had a trend of better survival than that with SLFN11^low^ tumors in both stage II (Fig. 4a) and stage III CRC, more prominent in stage III (100 % vs 72 %, Log-rank *P* = 0.092, Fig. 4b). NRAS and BRAF mutation was tested in KRAS exon 2 wild type patients, 10 and 3 patients were identified as mutation respectively. The results remained the same after exclude the 13 patients (data not shown).

**Table 2:** Univariate survival analysis based on KRAS status or SLFN11 expression status

			DFS			OS		
		No.	3y-DFS(%)	HR(95 %CI)	P	5y-OS	HR(95 %CI)	P
Patients with KRAS wt								
	SLFN11^high^	23	91.3	0.347(0.083–1.449)	0.147	100.0		0.05
	SLFN11^low^	130	75.8	1		83.3		
Patients with KRAS mt								
	SLFN11^high^	12	72.9	1.424(0.391–5.188)	0.592	80.2	1.348(0.279–6.508)	0.71
	SLFN11^low^	51	83.9	1		81.9	1	
Patients with SLFN11^high^								
	KRAS wt	23	91.3	1	0.207	100.0		0.06
	KRAS mt	12	72.9	3.167(0.528–18.979)		80.2		
Patients with SLFN11^low^								
	KRAS wt	130	75.8	1	0.511	80.5		0.503
	KRAS mt	51	83.9	0.787(0.386–1.607)		81.9	0.749(0.321–1.746)

## Discussion

The Schlafen (SLFN) family of proteins (from the German word *Schlieffen*, “sleeping”) includes several mouse and human members. Emerging evidence associates Schlafen proteins with control of cell proliferation, induction of immune responses, and regulation of viral replication. The family shows great diversity, with 10 murine and 5 human isoforms. This multigene family is reportedly comprised of three groups, which can be classified by the size of the encoded proteins [1, 4–6].

All SLFN proteins share a highly conserved N-terminal domain (AAA) that is involved in ATP/GTP binding [7]; however, only group III SLFNs, including SLFN11, harbor a motif found in superfamily I DNA/RNA helicases. Stable knockdown of *SLFNs* in malignant melanoma cells resulted in gain of stem-like properties, such as increased anchorage-independent growth, as evidenced by enhanced colony formation in soft agar assays [8]. In the intestinal mucosa, SLFN 3 helps regulate intestinal epithelial differentiation [9, 10] and affects intestinal development and maturation [11]. SLFN 3 exhibits antiproliferative properties, as evidenced by the fact that ectopic expression of SLFN 3 in human colon cancer cells significantly decreases proliferation [12]. In the current study, we found significant correlation between SLFN11 high expression and tumor differentiation. Poor or differentiated tumors tended not to express SLFN11, which accords with the ability of group III SLFNs to lead cells to differentiate and mature. SLFN11 could be a differentiation marker for CRC cells, and might be useful in stem cell separation.

DNA-damaging agents (DDAs) are a mainstay of treatment for most human tumors [13]. Both oxaliplatin and 5-FU are DDAs. The FOLFOX regimen is based on oxaliplatin and 5-FU, and is widely used in treating CRCs in both adjuvant and palliative settings. Zoppoli et al. showed SLFN11 expression had an extremely significant positive correlation with response to Top1 inhibitors (topotecan and irinotecan), Top2 inhibitors (doxorubicin, mitoxantrone, etoposide), DNA alkylating agents (chlorambucil, melphalan, cisplatin), and DNA synthesis inhibitors (gemcitabine and fludarabine) [3]. They also found that SLFN11 is causative in determining cell death and cell cycle arrest in response to DDAs in cancer cells that originate from different tissue types [3]. SLFN11 is a determinant of the cellular responses to DNA damage [3]. High SLFN11 expression independently predicted OS in a group of ovarian cancer patients treated with cisplatin-containing regimens [3]. Colon adenocarcinoma samples can have a range of SLFN11 expression to >600 % of the smallest level. SLFN11 expression could be a biomarker for response to adjuvant FOLFOX treatment of CRC. The current study is the first to report that FOLFOX sensitivity in SLFN11^high^ tumors was better than that in SLFN11^low^ tumors, but the effect was confined to *KRAS exon 2 wild-type* patients. Because all patients in this cohort received oxalipatin-based treatment, SLFN11 could be considered as both a prognostic and predictive factor in KRAS wt subgroup.

To the best of our knowledge, this is the first study that directly correlates SLFN11 expression in IHC staining with survival and other clinicopathological factors in CRC. SLFN11 tends to not express in poor differentiation cancers and our results imply that it can sensitize CRC cells to respond to oxaliplatin-based treatment. These findings provide valuable new information that advances our understanding of oxaliplatin based therapy in the adjuvant setting for patients with CRC. The precise mechanism by which SLFN11 sensitizes *KRAS* exon 2 CRC cells remains to be established. Stratifying patients with CRC by SLFN11 high or low expression may improve management and treatment of this disease and improve overall survival. Moreover, these findings raise the possibility of a strategy to enhance antineoplastic effects of some agents by developing drugs that promote SLFN11 expression.

The limitations of the study are as follows. First, this was a single center retrospective study. Second, the population of SLFN11 high expression patients was small. Therefore, prospective studies are warranted to verify the role of SLFN11 in all RAS wild-type patients.

## Conclusions

SLFN11 expression predicts good better survival in colorectal cancer patients with KRAS exon 2 wild type who have received oxaliplatin based adjuvant chemotherapy.
